# Spontaneous Remission of an Untreated, MYC and BCL2 Coexpressing, High-Grade B-Cell Lymphoma: A Case Report and Literature Review

**DOI:** 10.1155/2017/2676254

**Published:** 2017-02-21

**Authors:** D. Alan Potts, Jonathan R. Fromm, Ajay K. Gopal, Ryan D. Cassaday

**Affiliations:** ^1^University of Washington School of Medicine, 1959 NE Pacific Street, Seattle, WA 98195, USA; ^2^Department of Laboratory Medicine, University of Washington School of Medicine, P.O. Box 357110, 1959 NE Pacific Street, Seattle, WA 98195, USA; ^3^Department of Medicine, University of Washington School of Medicine, P.O. Box 356420, 1959 NE Pacific Street, Seattle, WA 98195, USA; ^4^Clinical Research Division, Fred Hutchinson Cancer Research Center, 1100 Fairview Avenue N, Seattle, WA 98109, USA

## Abstract

Non-Hodgkin lymphomas (NHL) are a heterogeneous group of hematologic malignancies typically treated with multiagent chemotherapy. Rarely, spontaneous remissions can be observed, particularly in more indolent subtypes. The prognosis of aggressive NHL can be predicted using clinical and histopathologic factors. In aggressive B-cell NHL, the importance of MYC and BCL2 proto-oncogene coexpression (as assessed by immunohistochemistry) and high-grade histologic features are particularly noteworthy. We report a unique case of spontaneous remission in a patient with an aggressive B-cell NHL which harbored high-risk histopathologic features, including MYC protein expression at 70–80%, BCL2 protein expression, and morphologic features suggestive of high-grade B-cell lymphoma, NOS (formerly B-cell lymphoma unclassifiable with features intermediate between diffuse large B-cell lymphoma and Burkitt lymphoma [BCLU]). After undergoing a biopsy to confirm this diagnosis, he opted to forego curative-intent chemotherapy. The single, yet relatively large area of involvement noted on ^18^F-fluorodeoxyglucose positron emission tomography-computed tomography steadily resolved on subsequent follow-up studies. He remained without evidence of recurrence one year later, having never received treatment. This case emphasizes the potential for spontaneous remission in NHL and demonstrates that this phenomenon can be observed despite contemporary high-risk histopathologic features.

## 1. Introduction

Non-Hodgkin lymphomas (NHL) are a heterogeneous group of hematologic malignancies that typically require multiagent cytotoxic chemotherapy and demonstrate a broad range of outcomes. Treatment of B-cell NHL typically relies upon multiagent cytotoxic chemotherapy (e.g., cyclophosphamide, doxorubicin, vincristine, and prednisone [CHOP]), tailored to the specific subtype of NHL and certain patient characteristics. With the addition of rituximab (R), a chimeric monoclonal antibody targeting the B-cell antigen CD20, outcomes have improved broadly for patients with B-cell NHL [1, 2]. Despite these improvements, however, many patients will ultimately suffer failure of this treatment.

A patient's risk of a poor outcome from a B-cell NHL can be assessed using several prognostic factors. Clinical determinants including age, extent of disease (according to the Ann Arbor staging system), presence of extranodal involvement, elevated lactate dehydrogenase (LDH), and performance status comprise the International Prognostic Index (IPI) [3]. Recently, certain histopathologic features have emerged as prognostic factors in aggressive B-cell NHL, the most common subtype of which is diffuse large B-cell lymphoma, not otherwise specified (DLBCL, NOS). The* myc* oncogene has prognostic significance when its expression is altered by gene rearrangement, as these often involve the promoter regions of immunoglobulin heavy or light chain genes, leading to overexpression in and proliferation of malignant B-cells [4–6]. In one study, patients with increased MYC protein expression, particularly when coupled with high expression of the antiapoptotic protein BCL2 as assessed by immunohistochemistry (IHC), experienced a median overall survival of only 3-4 years despite receiving curative-intent chemotherapy with R-CHOP [7]. By utilizing tools beyond routine histologic classification, clinicians can consider alternative treatment approaches for patients, particularly given these historically poor results with standard treatment.

Rarely, spontaneous regression is observed in patients diagnosed with NHL. The frequency of this phenomenon in all cancers has been reported separately as 1 case per 80,000–100,000 [8]. While not uncommon in low-grade lymphomas, spontaneous remission in aggressive, high-grade NHL is rare [9]. To our knowledge, it has not previously been described in the context of these more contemporary histopathologic risk-stratification systems. Here, we report a case of spontaneous remission in a patient with aggressive B-cell lymphoma harboring high-grade histologic features and MYC/BCL2 coexpression by IHC.

## 2. Case Presentation

**Figure 1:**
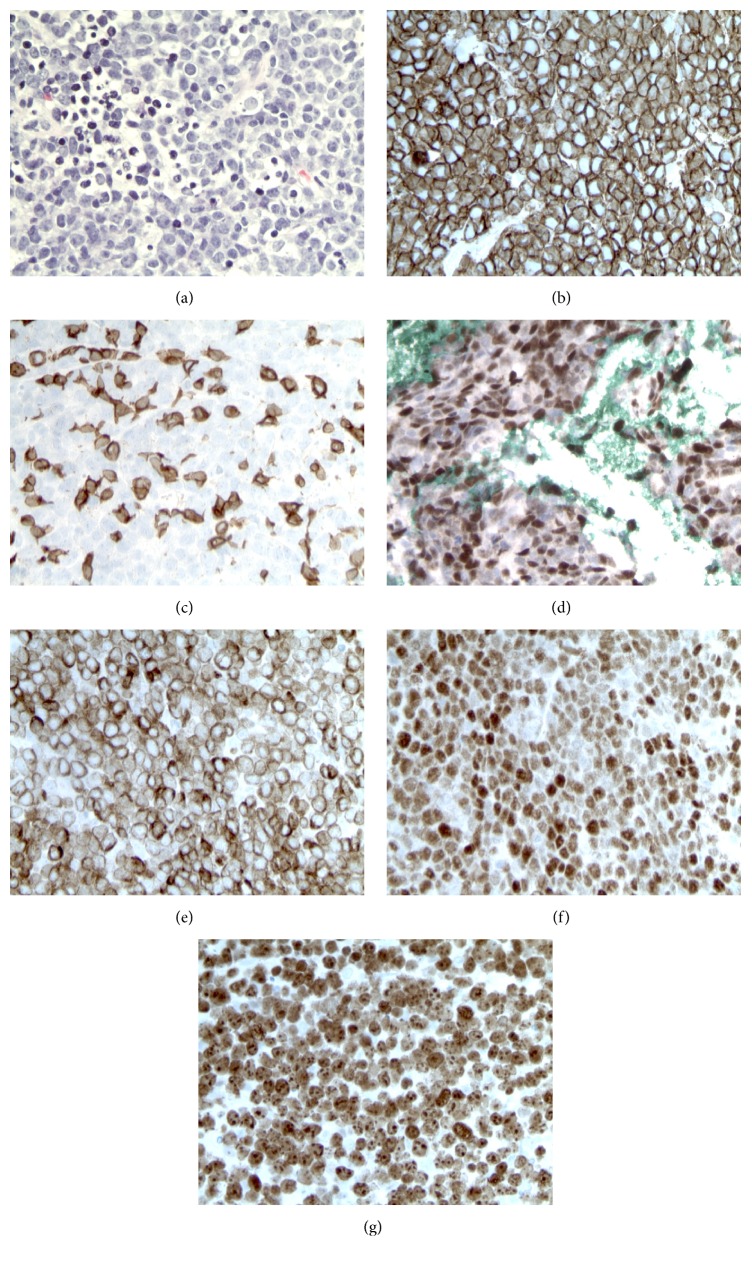
Photomicrographs of the needle core biopsy of the conglomerate periaortic lymph node mass. (a) Neoplastic proliferation of large, atypical lymphocytes (hematoxylin and eosin). Immunohistochemical analysis showed that the neoplastic cells were (b) uniformly positive for CD20 but (c) negative for CD3 (staining observed on small reactive T-cells). (d) MYC was positive in 70–80% of cells. (e) BCL2 and (f) BCL6 were both uniformly positive. (g) Ki-67 was positive in greater than 95% of cells. Images were captured using a Leica DM1000 microscope with digital camera using the manufacturer's FireCam software (Leica Microsystems, Bannockburn, IL). Original magnification, 640x.

A 59-year-old man with no significant medical history presented with three weeks of abdominal pain and weight loss. Computed tomography (CT) revealed conglomerate lymph nodes (LNs) surrounding the abdominal aorta measuring 5 × 6 cm, suspicious for lymphoma (Figure 1(a)). Laboratory data were largely unrevealing, including white blood cell count 8000/*μ*L with a normal differential, hemoglobin 15.8 g/dL, platelets 201,000/*μ*L, and normal assessments of renal and hepatic function. LDH was elevated at 209 units/L (laboratory normal range = 80–190 units/L). Human immunodeficiency virus (HIV) and hepatitis B serologies were unremarkable. CT-guided needle core biopsy, flow cytometry, and fluorescence in situ hybridization (FISH) studies were performed. Histologic sections of the para-aortic LN conglomerate demonstrated a neoplastic proliferation of large, atypical lymphocytes, arranged in sheets and characterized by intermediate to large-sized nuclei, dense finely granular chromatin, occasionally prominent nucleoli, and sparse cytoplasm. Numerous mitotic figures and apoptotic bodies were identified. By IHC, the neoplastic cells were uniformly positive for CD20 with intermixed positivity for CD3 on small lymphocytes. MYC (70–80% positive), BCL2 (greater than 95% positive), and BCL6 (greater than 95% positive) proteins were strongly expressed. The nuclear proliferative index (Ki-67) was markedly elevated (greater than 95%) (Figure 1). Flow cytometry demonstrated an abnormal CD10-positive B-cell population expressing CD19, CD20, and CD38 (increased) with lambda surface light chain expression without CD5, which comprised 70% of the white blood cells. Tumor cells were negative for MYC, BCL2, and BCL6 rearrangements by FISH. These results prompted the referring institution to diagnose high-grade B-cell lymphoma, not otherwise specified (HGBL) [10]. Consultative review by our center's hematopathologists agreed with high-grade B-cell NHL but provided a differential diagnosis of DLBCL, NOS, or HGBL.

**Figure 2:**
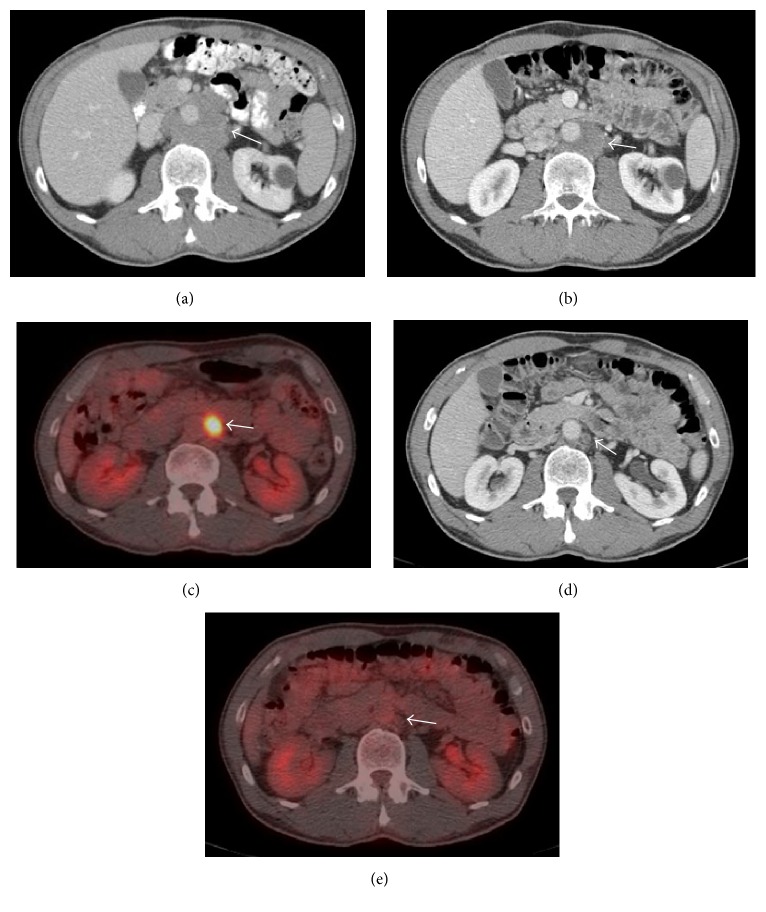
Representative images from serial computed tomography (CT) and ^18^F-fluorodeoxyglucose- (FDG-) positron emission tomography (PET) scans showing resolution of the biopsy-proven aggressive B-cell non-Hodgkin lymphoma in a conglomeration of periaortic lymph nodes. (a) CT image prior to diagnosis. (b and c) CT and FDG-PET images (resp.) at time of diagnosis showing interval decrease in size of the mass. (d and e) CT and FDG-PET images (resp.) six months after initial diagnosis, showing resolution of the mass. Arrows denote the location of the mass.

Surprisingly, within two weeks of biopsy, the patient's pain resolved. An ^18^F-fluorodeoxyglucose (FDG) positron emission tomography- (PET-) CT scan at that time noted intense avidity only in the para-aortic region (maximum standard uptake value of 17.4; Deauville score of 5), consistent with Stage I disease (Figures 2(b) and 2(c)). Interestingly, the LN size had decreased from the prior CT to 2.5 × 2.5 cm. His LDH had also fallen into the normal range (155 units/L). Against initial recommendations, the patient declined chemotherapy. He was followed with routine clinical assessments and did well despite foregoing treatment. Approximately six months after his initial diagnosis, a follow-up CT measured the LNs at 1.7 × 1.4 cm and was notable for the absence of the previously visualized hypermetabolic periaortic mass on FDG-PET (Figures 1(d) and 1(e)). Based on a Deauville score of 1, this would be classified as a complete response [11]. Laboratory results one year after his diagnosis were within normal limits. He had no symptoms or signs clinically to suggest recurrence.

## 3. Discussion

Spontaneous remission in aggressive lymphoma is a rare phenomenon with limited documentation in the medical literature. In a case series of NHL, Gattiker et al. found only 2 cases of spontaneous remission in 69 patients with diffuse lymphoma (2.9%) compared with 18 cases out of 140 in follicular lymphoma (12.9%) [12]. Notably, this report included patients with relapsed or stable disease following treatment, whereas our patient was treatment-naïve and demonstrated complete disappearance by PET-CT. Krikorian et al. studied 44 patients with advanced disease, and 6 (13.6%) showed remission without any previous treatment [13]. Clearly, the potential for spontaneous remission is evident in these cases. However, with the limited testing available at the time of these publications, the only apparent common thread linking those who underwent remission without therapy was a surgical biopsy of a peripheral lymph node performed for diagnostic purposes.

We present, to the best of our knowledge, the first reported case of spontaneous remission in an aggressive NHL harboring the poor prognostic features of MYC and BCL2 protein coexpression. With the improvements in IHC and other molecular and genetic testing, it is certainly feasible that prior reported cases of spontaneous remission in HGBL and DLBCL may have also demonstrated MYC and BCL2 coexpression that went undiscovered. Additionally, since the standard approach to a newly diagnosed, high-grade lymphoma is immediate intervention with aggressive chemotherapy, this treatment paradigm may have precluded other cases with the potential for spontaneous remission.

Complete remission in our patient is most remarkable, considering the expected poor prognosis associated with the protein overexpression (BCL2 and MYC) and cellular populations identified in his disease. Despite clinically favorable features (i.e., limited-stage disease, low-risk IPI score of 1), negative FISH for translocations in MYC, BCL2, or BCL6 (thereby avoiding the so-called “double-hit” or “triple-hit” lymphoma, which has been repeatedly associated with poorer outcomes in NHL patients), and a germinal center B-cell (GCB) subtype lymphoma as determined by the Hans algorithm (CD10 positive) (felt to represent a more favorable prognostic feature), the immunohistochemical features predicted a much worse outcome [4, 14–16]. Coexpression of the MYC and BCL2 proteins in DLBCL, newly defined as double-expressor DLBCL (DE DLBCL), is associated with significantly poorer overall survival (OS) and progression-free survival in patients who received curative-intent chemotherapy with R-CHOP compared to patients without coexpression [7, 10, 17]. Another retrospective analysis noted similar poor survival amongst patients with DE DLBCL despite dose-intensified therapy with R-double-CHOP [18]. Additionally, HGBL has a poorer outcome with standard treatment for DLBCL (i.e., R-CHOP), at least based on small retrospective analyses [4, 19]. One report noted a 23% 5-year event-free survival and 30% 5-year OS with treatment, far lower than the typical survival statistics seen in DLBCL [19]. As an uncommon and relatively newly described subtype of NHL, there have been no prospective randomized controlled trials specifically for HGBL. Therefore, the optimal treatment approach for this entity remains unclear.

The physiology of spontaneous remission remains unknown, although several proposed mechanisms exist. One leading hypothesis involves stimulation of an antitumor response from the host immune system, particularly immune activation secondary to infection or injury. Activation of antitumor immunity subsequent to local tissue injury and inflammation from a biopsy has been suggested previously in the literature, although the temporal relationship between intervention and decreasing lymph node size (as in our patient) may simply be coincidental [20, 21]. Spontaneous remission has also been frequently observed in patients with concomitant viral infections, such as human immunodeficiency virus and Epstein-Barr virus [22–24]. Still other cases support the immune system hypothesis by noting spontaneous remission of Hodgkin lymphoma after cessation of immunosuppressive therapy for autoimmune disease [25]. Importantly, our patient was not taking medications other than vitamin supplements and provided no subjective reports of illness during diagnosis and regression, although a subclinical infection would certainly be a possibility in a subject with no reported immunocompromise prior to his diagnosis.

The potential processes behind immunologic stimuli inducing remission are many and varied, but a unifying thought involves the immune system gaining the ability to recognize and react to malignant cells which were otherwise able to evade recognition. Therefore, any insult that activates host immunity theoretically enhances the possibility of host recognition of malignant cells. One mechanism for evading T-cell mediated cytotoxicity receiving extensive attention is expression of inhibitory ligands of immune checkpoint receptors (e.g., programmed death-1 [PD-1] and programmed death ligand [PD-L1]). Binding of these ligands on immune cells promotes T-cell receptor attenuation, which results in protection of the target tissue (the malignant cell, in this case) [26]. Monoclonal antibodies used pharmacologically against proteins involved in these pathways (e.g., ipilimumab [anti-CTLA4], pembrolizumab [anti-PD-1], and nivolumab [anti-PD-1]) have shown signs of efficacy against NHL in early clinical studies [27]. Unfortunately, due to the limited amount of tissue available from our patient's biopsy, we could not assay for these markers as a way to support these pathways as the mechanism for this spontaneous remission.

In summary, spontaneous remission of an aggressive NHL is a rare phenomenon with limited documentation in the medical literature. Complete remission without any specific intervention has not previously been reported in a patient like ours with such poor prognostic histopathologic features. Due to the expected poor outcome generally for patients with these characteristics, it is inadvisable to defer treatment in hopes of observing spontaneous remission in similar patients, as this aggressive disease may progress rapidly. However, the fact that spontaneous remission occurs in an aggressive, high-grade disease that historically is expected to respond poorly to intense chemotherapy is encouraging for the development of novel immunotherapeutic agents. Hopefully the intricacies of biology behind spontaneous remission in cancer (including aggressive lymphoma) will be uncovered, which may shed light on new therapeutic tools to improve the outcomes for patients afflicted with this disease.
